# Obesity influences the development of bisphosphonate-induced osteonecrosis in Wistar rats

**DOI:** 10.1590/1678-7757-2023-0133

**Published:** 2023-09-25

**Authors:** Wilson José de Miranda LIMA, Jannerson Cesar Xavier de PONTES, Ludmila Silva de FIGUEIREDO, Rubens da Silva ARAÚJO, Maria Carolina de PAIVA SOUSA, Jailane de Souza AQUINO, Ricardo Dias de CASTRO, Adriano Francisco ALVES

**Affiliations:** 1 Universidade Federal da Paraíba Centro de Ciências da Saúde Programa de Pós-Graduação em Odontologia João Pessoa Paraíba Brasil Universidade Federal da Paraíba, Centro de Ciências da Saúde, Programa de Pós-Graduação em Odontologia, João Pessoa, Paraíba, Brasil.; 2 Universidade Federal da Paraíba Centro de Ciências da Saúde Programa de Pós-Graduação em Produtos Naturais e Sintéticos Bioativos João Pessoa Paraíba Brasil Universidade Federal da Paraíba, Centro de Ciências da Saúde, Programa de Pós-Graduação em Produtos Naturais e Sintéticos Bioativos, João Pessoa, Paraíba, Brasil.; 3 Universidade Federal da Paraíba Centro de Ciências da Saúde Faculdade de Odontologia João Pessoa Paraíba Brasil Universidade Federal da Paraíba, Centro de Ciências da Saúde, Faculdade de Odontologia,João Pessoa, Paraíba, Brasil.; 4 Universidade Federal da Paraíba Centro de Ciências da Saúde Programa de Pós-Graduação em Ciências da Nutrição João Pessoa Paraíba Brasil Universidade Federal da Paraíba, Centro de Ciências da Saúde, Programa de Pós-Graduação em Ciências da Nutrição,João Pessoa, Paraíba, Brasil.; 5 Universidade Federal da Paraíba Centro de Ciências da Saúde Departamento de Clínica e Odontologia Social João Pessoa Paraíba Brasil Universidade Federal da Paraíba, Centro de Ciências da Saúde, Departamento de Clínica e Odontologia Social, João Pessoa, Paraíba, Brasil.; 6 Universidade Federal da Paraíba Centro de Ciências da Saúde Departamento de Fisiologia e Patologia, João Pessoa Paraíba Brasil Universidade Federal da Paraíba, Centro de Ciências da Saúde, Departamento de Fisiologia e Patologia, João Pessoa, Paraíba, Brasil.

**Keywords:** Osteonecrosis, Diphosphonates, Obesity, Mandible, Rats, Wistar

## Abstract

Medication-related osteonecrosis of the jaw (MRONJ) is characterized by bone exposure for more than eight weeks in patients who have used or been treated with antiresorptive or antiangiogenic drugs, without a history of radiation therapy or metastatic diseases in the jaws. Obesity is associated with changes in periodontal tissues and oral microbiota that are linked to bone alterations. This study aimed to analyze the influence of obesity on the development of bisphosphonate-induced osteonecrosis. The experiment randomly and simply divided 24 male Wistar rats (Rattus norvegicus) into four groups: healthy, with osteonecrosis, obese, and obese with osteonecrosis (n=6 per group). Osteonecrosis was induced through weekly intraperitoneal injection for eight weeks at a dose of 250 µg/kg of zoledronic acid in a 4 mg/5 mL solution, combined with trauma (exodontia). Obesity was induced through a high glycaemic index diet. Each group was qualitatively and quantitatively evaluated regarding the development of models and pathological anatomy of the lesions. The results were expressed in mean percentage and standard deviation and statistically analyzed using one-way analysis of variance (ANOVA) followed by Tukey's post-hoc test, with a significance level of 5% (p<0.05) to establish differences found between the groups. Animals in the osteonecrosis group and the obese with osteonecrosis group presented larger necrosis areas (averages: 172.83±18,19 µm^2^ and 290.33±15,77 µm^2^, respectively) (p<0,0001). Bone sequestration, hepatic steatosis, and increased adipocyte size were observed in the obese group (average: 97.75±1.91 µm^2^) and in the obese with osteonecrosis group (average: 98.41±1.56 µm^2^), indicating greater tissue damage in these groups (p<0,0001). All parameters analyzed (through histological, morphometric, and murinometric analyses) increased for the obese and obese with osteonecrosis groups, suggesting a possible influence of obesity on the results. However, further studies are needed to confirm the role of obesity in the possible exacerbation of osteonecrosis and understand the underlying mechanisms.

## Introduction

The American Association of Oral and Maxillofacial Surgeons (AAOMS) has defined medication-related osteonecrosis of the jaws (MRONJ) as the presence of bone exposure or bone that can be probed through an intraoral or extraoral fistula in the maxillofacial region, persisting without repair for more than eight weeks in patients currently under or previously treated with antiresorptive or antiangiogenic drugs, without prior radiation therapy or metastatic diseases in the jaws.^1^

Among the antiangiogenic and antiresorptive drugs with the potential to induce the disease, we can mention bisphosphonates, which belong to a class of medications indicated for the treatment of osteolytic disorders or bone metabolism disorders such as osteoporosis, Paget’s disease, bone metastases (mainly resulting from primary tumors located in the breast, lung, prostate, or multiple myeloma), malignancy-associated hypercalcemia, and osteogenesis imperfecta.^2^

Bisphosphonates act by inhibiting bone resorption, mainly because of their inhibitory action on osteoclasts. They concentrate in bones, due to their high affinity for hydroxyapatite crystals. During osteoclastic resorption, these drugs are phagocytosed by osteoclasts along with the degraded bone matrix through the action of proteases and acids released by these cells. Once phagocytosed, bisphosphonates act within osteoclasts, impairing their functioning.^3^

Obesity is a significant public health problem, affecting approximately 650 million adults worldwide. According to the World Health Organization (WHO), obesity results from the accumulation of fat in the body. The excess of adipose tissue leads to a state of chronic inflammation and oxidative stress, which cause cellular damage.^4^ Both situations are related to the development of comorbidities associated with obesity, including insulin resistance, dyslipidemia, hepatic steatosis, intestinal issues, renal diseases, hypertension, and the emergence of various types of cancer, such as colorectal cancer.^5^

Studies have addressed the relationship between obesity and bone changes in animals. Chaves, et al.^6^ (2022) investigated the impact of obesity on periodontal tissues and oral microbiota in mice and identified that obesity can induce alterations in the oral microbiota and neutrophil recruitment, which are associated with alveolar bone loss. Muluke, et al.^7^ (2016) conducted a study comparing high-fat diets to induce obesity and oral infection with Porphyromonas gingivalis to assess impact on bone tissue. They identified that obesity is associated with abnormal lipid metabolism and compromised bone homeostasis.

Many questions related to MRONJ still need to be clarified. Despite the existence of hypotheses that seek to understand the underlying mechanisms of osteonecrosis, a thorough study of the complex and dynamic mechanisms involving bone pathophysiology is still necessary for a better understanding of this disease.^8^ The present study hypothesized that chronic inflammatory diseases such as obesity influence the development of osteonecrosis. Based on this, its objective was to morphologically analyze the influence of obesity on the experimental development of bisphosphonate-induced osteonecrosis in an animal model.

## Methodology

### Sample

The experiment included 24 male Wistar rats (*Rattus norvegicus*) aged approximately 30 days and weighing 180 g. They were simply and randomly divided into four groups (using computer-generated randomization): healthy (1), with osteonecrosis (2), obese (3) and obese with osteonecrosis (4), (n=6 per group). The animals were kept in the vivarium of the Research Institute for Pharmaceuticals and Medicines (IPeFarM) and Laboratory of Experimental Nutrition (LANEX) of the Federal University of Paraíba. The study is filed with the Commission for Ethics in the Use of Animals (CEUA) under number 8738120220 (ID 000661) (attachments 01 and 02), in accordance with the precepts of Law 11,794 of October 8, 2008, Decree 6,899 of July 15, 2009, and the rules issued by the National Council for the Control of Animal Experimentation (CONCEA).

Based on the data described in the literature,^9^ we expected a population variability (standard deviation) of around 30% and a 50% (mean) difference between the experimental groups for the main experimental parameter. Using one-way analysis of variance (ANOVA) followed by Tukey’s post-test comparison with alpha values (type I error - 5%) and beta values (type II error - 80%), we required 5 animals per group (n=5). Considering a mortality rate of 15% or procedural issues, we needed one additional animal per group, totalling 6 animals. The study consisted of four groups, which justifies the total sample size of 24 animals. The calculation was performed through the statistics website of the University of São Paulo.

### Induction of osteonecrosis

#### Induction of osteonecrosis for groups 1 and 2

**Figure 1:**
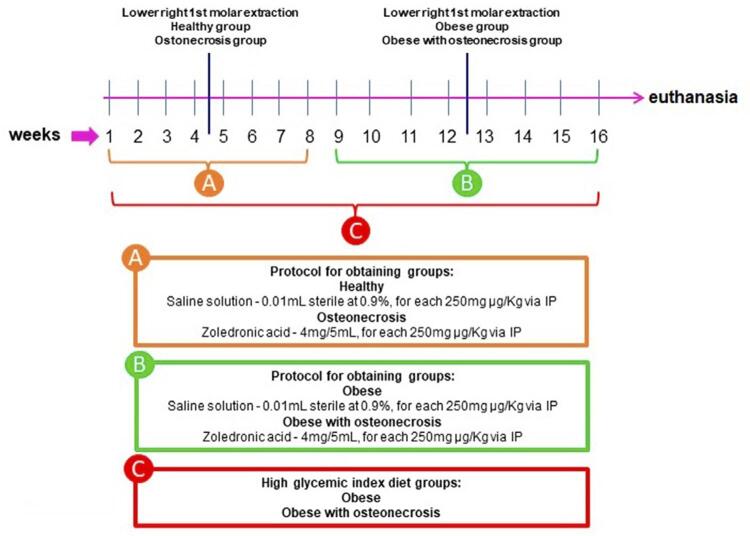
(A) Weekly details of the osteonecrosis induction protocol to obtain groups 1 and 2. (B) and (C) Weekly details of the osteonecrosis induction protocol to obtain groups 3 and 4

Osteonecrosis was induced by administrating zoledronic acid (bisphosphonate), adapting the methodology of Biguetti, et al.^10^ (2019). The induction was associated with the extraction of the lower left first molar. The animals in the healthy group (1) received saline intraperitoneally, while the ones in the osteonecrosis group (2) received 250 µg/kg of zoledronic acid (4mg/5ml; Blau Farmaceutica) intraperitoneally, weekly, for eight weeks (Figure 1A).^10^

Between the administration of the fourth and fifth doses of the drug, all animals underwent the extraction of their lower left first molar. The animals were sedated with an association of ketamine (100 mg/kg) and xylazine (30 mg/kg) injected intraperitoneally, ensuring immobilization and general anesthesia between 30 and 45 minutes.^11^ A number five exploratory probe was used to dislocate the tooth.

#### Induction of osteonecrosis for groups 3 and 4

The induction of osteonecrosis in groups 3 and 4 was performed as described above but in a different period, because the animals had to go through the obesity induction protocol in parallel (see below), which lasted 16 weeks (Figure 1B and C).^10^

## Induction and evaluation of obesity

### Diet

**Figure 2:**
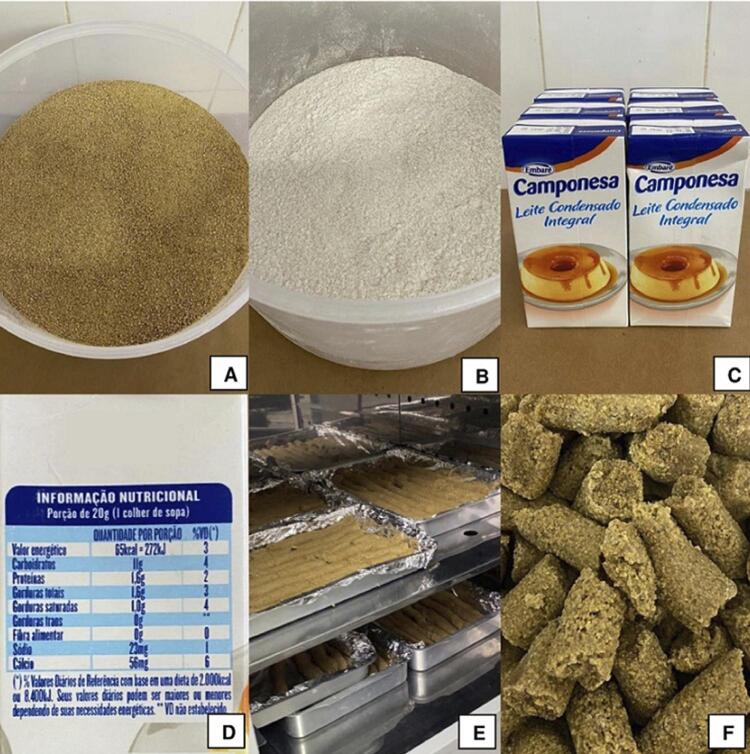
Feed preparation for obesity induction. A – Crushed and sifted feed; B – Refined and sifted sugar; C – Condensed milk used to prepare the feed; D – Nutritional information for condensed milk (20g serving - 1 tablespoon - 65kcal = 272kJ, DV(*) 3%; Carbohydrates 11g DV(*) 4%; Proteins 1,6g DV(*) 2%; Total fat 1,6g DV(*) 3%; Saturated fat 1,0g DV(*) 4%; Trans fats 0g **; Dietary Fiber 0g DV(*) 0%; Sodium 23mg DV(*) 1%; Calcium 56mg DV(*)6%; Daily Reference Values based on a 2.000kcal or 8.400kJ diet. Your daily values may be higher or lower depending on your energy needs. ** DV not established. ALLERGENS: CONTAINS MILK. CONTAINS LACTOSE. GLUTEN-FREE.); E – Molded feed placed in the oven for the drying step; F – Feed after drying ready for consumption

The animals in groups 3 and 4 (obese and obese with osteonecrosis) received a balanced diet composed of pellets (Nuvilab^®^), refined sugar (Alegre^®^), and whole condensed milk (Peasant^®^). This diet had a high glycemic index and a load of 77.6 and 38.8, respectively. For 100 g of the high glycemic index diet, 45.2 g of the ration was crushed and mixed with 9.6 g of sugar and 45.2 mL of condensed milk. The mixture was homogenized, molded, dried in an oven (55 °C) for 24 h, and stored under refrigeration. 800 g per week were available to the animals in pellets for 16 weeks (Figure 2) (adapted from Luz, et al.^12^ (2018)).

### Weight gain and food intake

The rats’ body mass (g) was checked once a week, and the mass gain was calculated by the difference between the final and initial body mass. Food consumption was checked once a week, always on the same day, and was represented by the difference between food offered and waste (consumption (g) = quota offered (g) – clean waste (g)). Clean waste was the non-ingested food that remained in the box.

### Naso-anal length, Lee index, and body mass index

**Figure 3:**
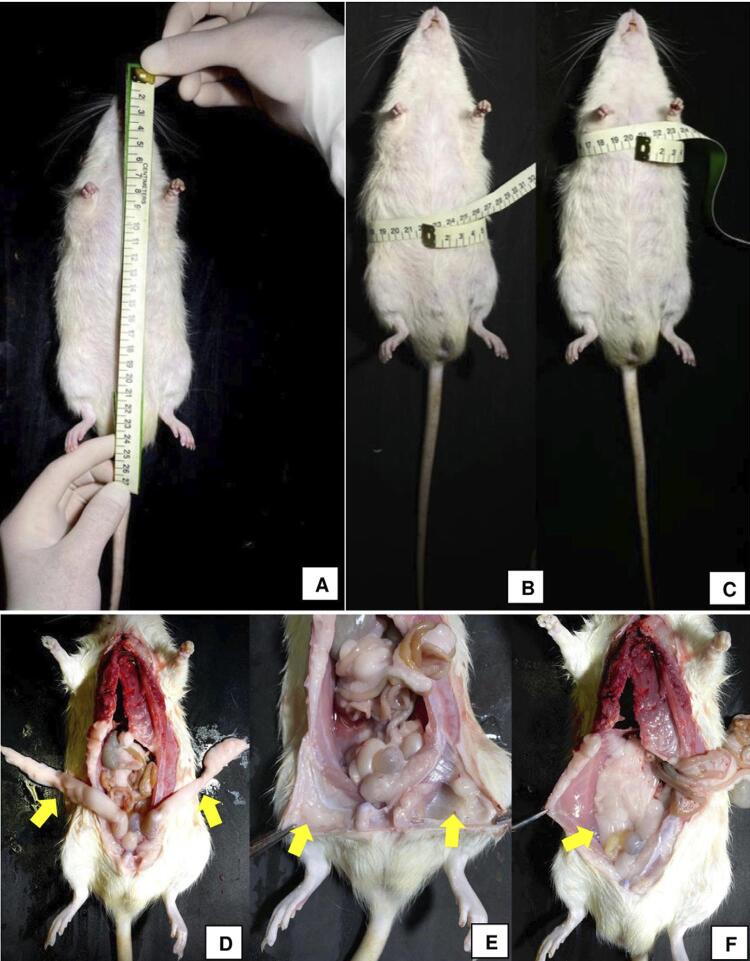
Body measurement protocol and regions where the adipose tissue was removed (yellow arrows). (A) - Naso-anal length measurement; (B) – Abdominal circumference measurement; (C) Chest circumference measurement; (D) – Epididymal adipose tissue; (E) – Inguinal adipose tissue; (F) – Retroperitoneal adipose tissue

On the day of euthanasia, the animals were weighed, and their naso-anal length (cm), which was used to calculate the Lee index, was measured (Figure 3). The Lee index is defined as the ratio between the animal’s body mass cube root and naso-anal length. The body mass index (BMI) was also calculated, characterized by the ratio between the animal’s weight and the squared naso-anal length.^13^

### Abdominal and chest circumferences

On the day of euthanasia, the circumference of the abdomen, located in front of the animal’s hind leg, and of the thorax, located in the posterior portion of the front leg, was measured with an anthropometric body tape (Figure 3).^13^

### Mass of adipose tissue depots, adiposity index, and liver weight

The adipose tissues of the inguinal, retroperitoneal, and epididymal regions, which are considered the main components of central adiposity in rats, were weighed. Epididymal fat is the deposit located in the lower portion of the abdomen, connected to the epididymis. Inguinal fat is located between the lower portion of the ribcage and the medial portion of the thigh. The fat connected to the posterior abdominal wall represents the retroperitoneal fat (Figure 3).^14^

The adiposity index was calculated from the sum of the individual masses of the epididymal, inguinal, and retroperitoneal adipose layers, multiplied by 100 and divided by the final body weight. However, the retroperitoneal region was used as a representative. Previous studies indicated that with eight weeks of a diet rich in refined carbohydrates, the rats already presented hypertrophic retroperitoneal adipose tissue.^15^ Finally, during euthanasia, the liver was dissected and weighed.

## Physical examination of the oral cavity

The physical examination was performed through direct observation in a room with appropriate lighting. Soft tissues were separated with tweezers, and a photographic record was made. Each animal was evaluated for the presence or absence of bone exposure: intra- or extra-oral fistula.

## Histological processing

After euthanasia and physical examination, each sample—composed, at that moment, of the mandible, fat, and liver—was fixed in formalin. After 48 hours of fixation, the mandibles were decalcified in a 5% nitric acid solution. After demineralization, the material underwent cleavage, along with the other samples—organs and fat—and was processed, undergoing dehydration in solution baths with increasing concentrations of ethyl alcohol, clarified in xylene, impregnated in paraffin baths (maximum temperature of 60 ºC), and embedded in paraffin in inclusion molds. The material then underwent microtomy (4 μm thickness) and was stained with hematoxylin-eosin.

## Histological analysis in light microscopy and histomorphometry

The cuts were observed and analyzed under a light microscope, evaluated, and photographed using the Nikon microscope software Model Eclipse Ci-L (Tokyo, Japan). They were qualitatively evaluated for the presence or absence of bone necrosis, bone sequestration, infection, periosteal reaction, and bone marrow adiposity. Histologically, osteonecrosis is defined by the presence of five or more adjacent empty osteoclasts.^16^

To calculate the bone necrosis area, histological sections were analyzed with a 40X objective and interactively quantified using the Image-Pro Plus software. The entire interstitium was excluded from the analysis using the program’s tools, and a binary image was created with the area calculated in µm^2^.

## Statistical analysis

The results were initially analyzed using descriptive statistics of the data, calculating the maximum and minimum values, amplitude, mean, and standard deviation. The Shapiro-Wilk normality test was performed, and the data was tabulated and analyzed based on analyses macroscopic and histomorphometric analyses were tabulated and analyzed using one-way analysis of variance (ANOVA) followed by Tukey’s post-test, with a statistical significance level of 5% (p<0.05) to establish the differences found between the experimental groups. All data were analyzed using the GraphPad Prism^®^ version 5.04 program.

## Results

### Induction and evaluation of obesity

#### Weight evolution

**Figure 4:**
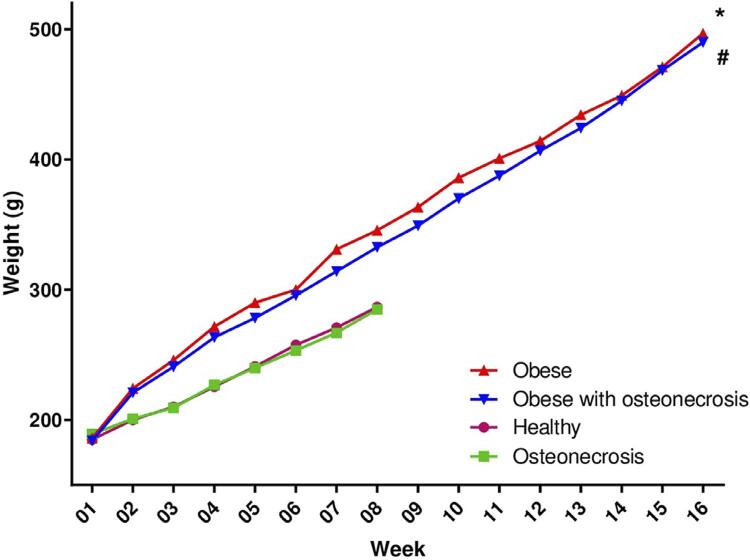
Weekly average body mass of the animals in each study group. Symbols and vertical lines represent the means (n=6). One-way ANOVA followed by Tukey's post-test. *p<0.05 (obese vs. healthy and obese vs. osteonecrosis), #p<0.05 (obese with osteonecrosis vs. healthy and obese with osteonecrosis vs. osteonecrosis)

The following means and standard deviations were found in the weight evolution: 350.6±92.1 g in the obese group, 341.9±91.4 g in the obese group with osteonecrosis, 234.6±35.7 g in the healthy group, and 233.9±33.5 g in the osteonecrosis group (Table 1). A greater weight evolution was observed in the obese groups after 16 weeks of consuming a high-glycemic index diet, with an increase in weight observed from the first weighing (Figure 4). Significant statistical differences were found between the means (p=0.0004).

**Table 1:** Means and standard deviations of the analyzed variables by group

Variable	Healthy	Osteonecrosis	Obese	Obese with osteonecrosis
Weight evolution	234.6 g ± 35.7 g	233.9 g ± 33.5 g	350.6 g ± 92.1 g	341.9 g ± 91.4 g
Naso-anal length	25.33 cm ± 0.51 cm	25.33 cm ± 0.51 cm	27.17 cm ± 1.16 cm	26.00 cm ± 1.09 cm
Lee index	0.26 g/cm ± 0.01 g/cm	0.25 g/cm ± 0.00 g/cm	0.30 g/cm ± 0.00 g/cm	0.30 g/cm ± 0.00 g/cm
Body mass index	0.44 g/cm^2^ ± 0.01 g/cm^2^	0.44 g/cm^2^ ± 0.03 g/cm^2^	0.68 g/cm^2^ ± 0.04 g/cm^2^	0.69 g/cm^2^ ± 0.04 g/cm^2^
Abdominal circumference	18.07 cm ± 0.38 cm	18.07 cm ± 0.44 cm	20.10 cm ± 0.21 cm	19.91 cm ± 0.14 cm
Chest circumference	16.67 cm ± 0.51 cm	16.50 cm ± 0.54 cm	18.08 cm ± 1.28 cm	15.92 cm ± 0.86 cm
Adiposity index	6.43 g ± 1.00 g	5.83 g ± 0.68 g	10.08 g ± 1.03 g	10.42 g ± 0.86 g
Liver weight	10.62 g ± 0.44 g	10.67 g ± 0.81 g	12.93 g ± 1.04 g	12.54 g ± 1.38 g

#### Naso-anal length, Lee index, and body mass index

**Figure 5:**
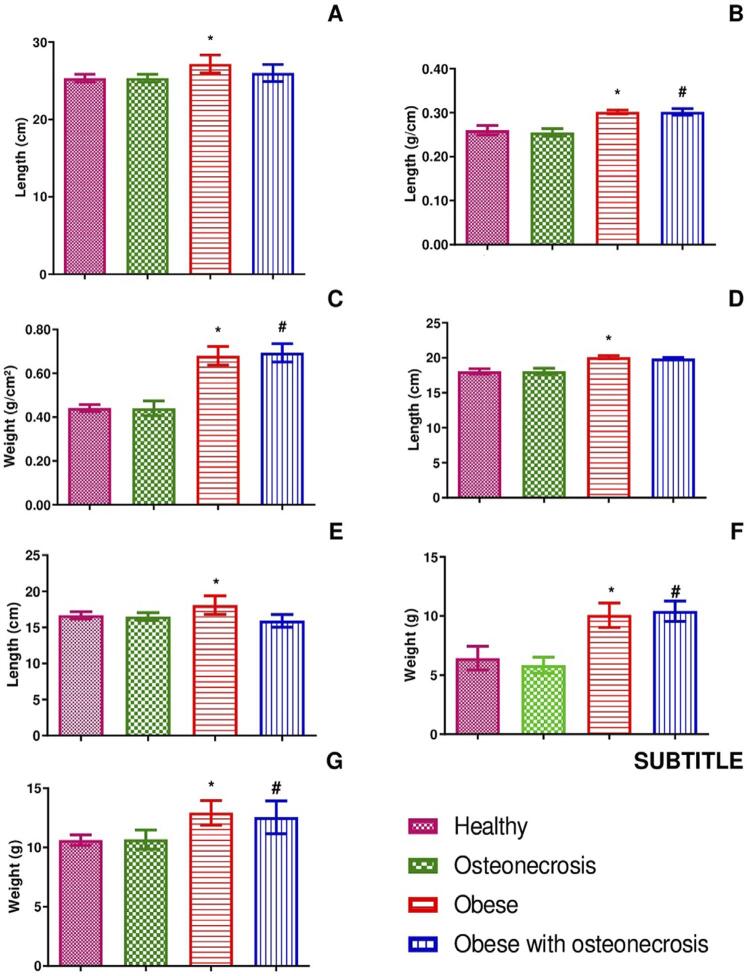
Result of the murinometric parameters evaluation. Symbols and vertical bars represent the means (n=6). One-way ANOVA followed by Tukey's post-test. *p<0.05 (obese vs. healthy and obese vs. osteonecrosis), #p<0.05 (obese with osteonecrosis vs. healthy and obese with osteonecrosis vs. osteonecrosis). (A) Naso-anal length; (B) Lee index; (C) Body mass index; (D) Abdominal circumference; (E) Thoracic circumference; (F) Adiposity index; (G) Liver weight

The means and standard deviations of the naso-anal length were higher in the obese group (27.17±1.16 cm), followed by the obese group with osteonecrosis (26.00±1.09 cm). Meanwhile, the healthy group and the osteonecrosis group had the same mean and standard deviation (25.33±0.51 cm) (Table 1). Significant statistical differences were found between the means (p=0.0051) (Figure 5A).

The following means and standard deviations were obtained with the Lee index: 0.30±0.00 g/cm in the obese group and obese group with osteonecrosis, 0.26±0.01 g/cm in the healthy group and 0.25±0.00 g/cm in the osteonecrosis group (Table 1). Significant statistical differences were found between the means (p<0.0001) (Figure 5B).

The following means and standard deviations of the body mass index were obtained: 0.69±0.04 g/cm^2^ in the obese group with osteonecrosis, 0.68±0.04 g/cm^2^ in the obese group, 0.44±0.01 g/cm^2^ in the healthy group, and 0.44±0.03 g/cm^2^ in the osteonecrosis group (Table 1). Significant statistical differences were found between the means (p<0.0001) (Figure 5C).

#### Abdominal and chest circumferences

The means and standard deviations of the abdominal circumference were 20.10±0.21 cm in the obese group, 19.91±0.14 cm in the obese group with osteonecrosis, 18.07±0.38 cm in the healthy group, and 18.07±0.44 cm in the osteonecrosis group (Table 1). Significant statistical differences were found when comparing the group means (p<0.0001) (Figure 5D).

For chest circumference, the following means and standard deviations were found: 18.08±1.28 cm in the obese group, 16.67±0.51 cm in the healthy group, 16.50±0.54 cm in the osteonecrosis group, and 15.92±0.86 cm in the obese group with osteonecrosis (Table 1). Significant statistical differences were found when comparing the group means (p=0.0023) (Figure 5E).

#### Adiposity index and liver weight

The mean values and standard deviations for the adiposity index were: 10.42±0.86 g in the obese group with osteonecrosis, 10.08±1.03 g in the obese group, 6.43±1.00 g in the healthy group, and 5.83 ± 0.68 g in the osteonecrosis group (Table 1). Significant statistical differences were found when comparing the group means (p<0.0001) (Figure 5F).

Regarding liver weight, the following means and standard deviations were obtained: 12.93±1.04 g in the obese group, 12.54±1.38 g in the obese group with osteonecrosis, 10.67±0.81 g in the osteonecrosis group, and 10.62±0.44 g in the healthy group (Table 1). Significant statistical differences were found when comparing the group means (p=0.0005) (Figure 5G).

## Anatomical pathology analysis and morphometry

**Figure 6:**
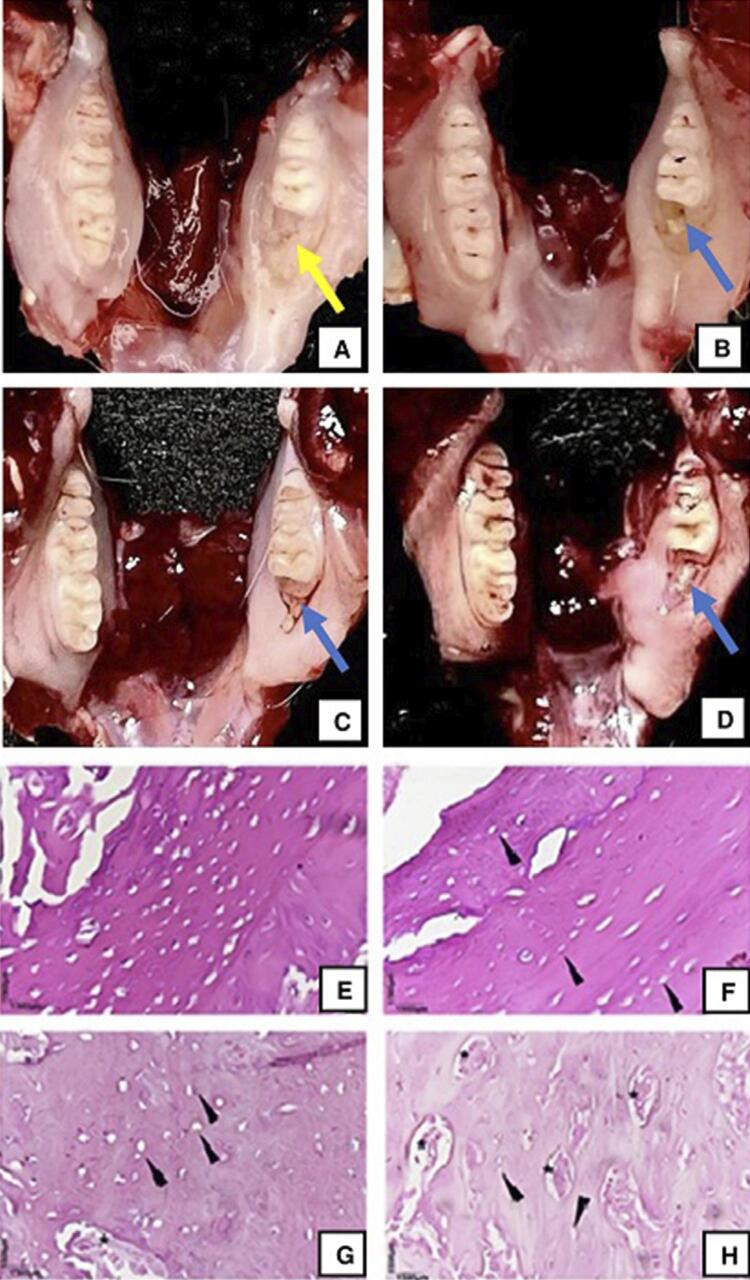
Representative macroscopy and microscopy of the animals’ dissected mandibles, per group. (A, E) - Healthy control; (B, F) – Osteonecrosis; (C, G) – Obese; (D, H) - Obese with osteonecrosis. Yellow arrow, region with gingival tissue covering the alveolar region. Blue arrow, region of exposed bone. Black arrowhead, osteonecrosis. * bone sequestration (400X magnification)

The macroscopic analysis of the mandible indicated that the animals in the healthy group exhibited clear, smooth, and homogeneous tissue in the extraction area, consistent with healthy gums (Figure 6A - yellow arrow). However, the animals in the osteonecrosis, obese, and obese with osteonecrosis groups showed an absence of epithelial lining in the tooth extraction region, which is evidenced by a macroscopically yellowish area indicative of exposed bone (Figure 6B, C, and D - blue arrow).

Microscopically, the healthy group (Figure 6E) presented bone tissue with nucleated osteocytes without morphological alterations. On the other hand, the osteonecrosis group (Figure 6F - black arrows) displayed anucleate osteocytes, compatible with osteonecrosis. Bone sequestration was also observed in the animals from the obese (Figure 6G - asterisks *) and obese with osteonecrosis (Figure 6G - asterisks *) groups.

**Figure 7:**
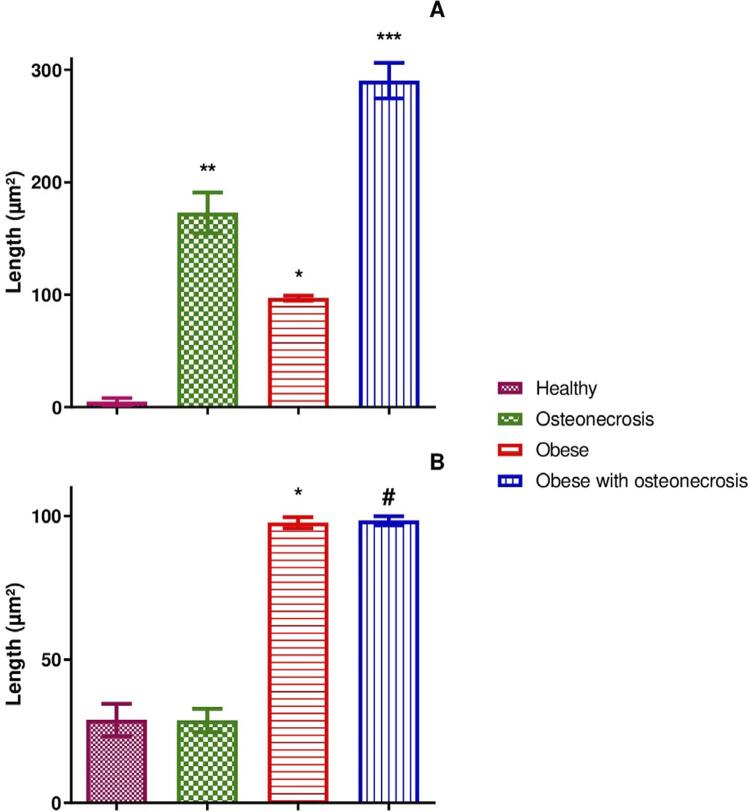
(A) - Mean areas of necrosis and (B) - Mean adipocyte size in animals from each study group. Symbols and vertical bars represent the means (n=6). One-way ANOVA followed by Tukey's post-test. In (A) - ***p<0.05 (obese vs. healthy), **p<0.05 (osteonecrosis vs. healthy) and *p<0.05 (obese vs. healthy). In (B) - *p<0.05 (obese vs. healthy and obese vs. osteonecrosis), #p<0.05 (obese with osteonecrosis vs. healthy and obese with osteonecrosis vs. osteonecrosis)

Based on the morphometric analysis results, the following means and standard deviations were obtained for the necrosis area: 290.33±15.77 µm^2^ in the obese with osteonecrosis group, 172.83±18.19 µm^2^ in the osteonecrosis group, 97.00±2.13 µm^2^ in the obese group, and 5.00±3.21 µm^2^ in the healthy group (Figure 7A). Significant statistical differences were found when comparing the group means p<0,0001).

**Figure 8:**
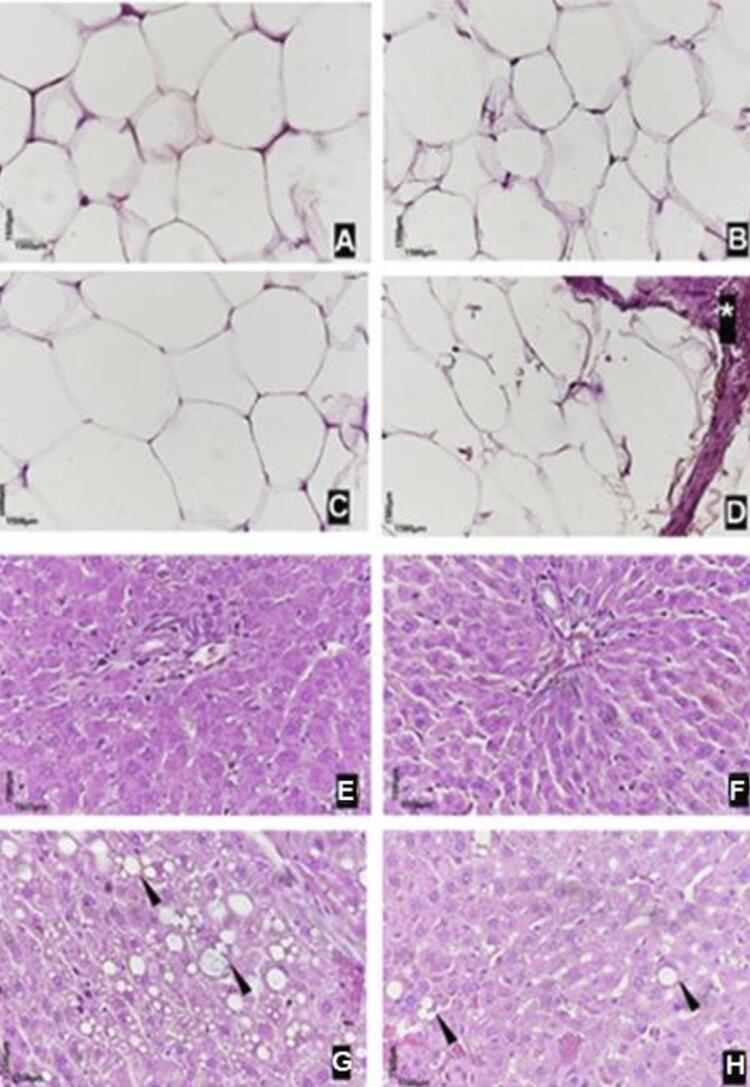
Representative adipose tissue microscopy: (A) - Healthy; (B) - Osteonecrosis; (C) – Obese; (D) – Obese with osteonecrosis. *blood stasis. Representative hepatic parenchyma microscopy: (E) – Healthy; (F) - Osteonecrosis; (G) – Obese; (F) - Obese with osteonecrosis. Black arrowhead, hepatic steatosis (400X magnification)

The microscopic evaluation of the adipose tissue (Figure 8) indicated that the adipocytes in the healthy (A) and osteonecrosis (B) groups did not undergo inflammatory processes. On the other hand, large adipocytes were observed in the animals in the obese (C) and obese with osteonecrosis (D) groups, and only the animals in the latter group presented dilated vessels, corresponding to blood stasis in the adipose tissue, indicated by an asterisk (*).

The microscopic evaluation of the liver (Figure 8) indicated a total preservation of its parenchyma in the animals of the healthy (E) and osteonecrosis (F) groups. In the animals from the obese (G) and obese with osteonecrosis (H) groups, the hepatic parenchyma displayed areas of steatosis (Figure 8 - G and H - arrowhead).

The morphometric analysis of the adipose tissue indicated the following means and standard deviations for adipocyte size: 98.41±1.56 µm^2^ in the obese with osteonecrosis group, 97.75±1.91 µm^2^ in the obese group, 28.91±5.68 µm^2^ in the healthy group, and 28.75±4.09 µm^2^ in the osteonecrosis group (Figure 7B). Significant statistical differences were found when comparing the group means (p<0,0001).

## Discussion

The present research implemented and standardized, in an isolated and associated manner, models of obesity induced by a high glycemic index diet (HGID) and osteonecrosis induced by bisphosphonates in Wistar rats. The obesity model was characterized by an increase in the animals’ final weight, adipocyte size, naso-anal length, thoracic and abdominal circumferences, and Lee index. Microscopic changes, such as the presence of empty osteoclasts, bone sequestration, and increased infiltration of inflammatory cells in the studied region, characterized osteonecrosis implementation. The combination of both conditions exacerbated some of the parameters observed in each individual condition.

In Brazil, estimates of obesity prevalence, according to the Telephone Survey of Risk and Protective Factors for Chronic Diseases, increased from 15% to 18% in both sexes from 2010 to 2014. In the Family Budget Survey (FBS), the prevalence of obesity among men increased from 9.3% (FBS 2002-2003) to 12.7% (FBS 2008-2009). Among women, the prevalence of obesity increased from 14.0% to 17.5% in the respective surveys. Thus, the importance of studies on this disease and its possible associations with other factors is highlighted.^17^

This study analyzed the influence of obesity on the development of bisphosphonate-induced osteonecrosis through an animal model capable of correlating these diseases. The creation of animal models of obesity arose mainly due to ethical limitations in studying the susceptibility of humans to pathological dysfunctions caused by obesity.^18^

Another challenge in studying this clinical alteration is the fact that it is a multifactorial disease, and the objective was to mimic the changes caused by human obesity in rodents. Currently, there are several proposed models for inducing obesity, and one of the main methods is through dietary alteration, a process that resembles what occurs in human obesity.^19^

The high glycemic index (HGID) diet chosen for the study includes condensed milk as one of its main components and was offered to the animals in the obese and obese with osteonecrosis groups for 16 weeks. Masi, et al.^20^ (2017) compared two diets in mice: a sugar-rich one and a fat-rich one. They reported an increase in body weight, along with other alterations, such as glucose intolerance, hepatic fibrogenesis, and increased inflammation in animals fed with a diet high in condensed milk, demonstrating that this ingredient is more inflammatory than fat.^20^

Ferreira, et al.^21^ (2022) conducted a study that linked asthma to obesity, also using a high glycemic index diet, and obtained similar results to those found in the present study: they identified an increase in body weight, fasting glucose, abdominal circumference, body mass index, and adiposity index, in addition to asthma exacerbation due to obesity.

In this study, the animals that received the HGID exhibited a greater weight gain compared to those that received the common diet (feed only), which is an important factor for characterizing obesity induction. Similar results were observed by Luz, et al.^12^ (2018) and by Ferreira, et al.^21^ (2022), who found that animals in the experimental groups gained weight in 16 weeks of a HGID diet. Ferreira, et al.^21^ (2022) carried out a study with an obesity-inducing diet similar to the one discussed above and found an increase in the animals’ naso-anal length, corroborating the data found in the present study. Similarly, Novelli, et al.^22^ (2007) submitted animals to a carbohydrate-rich diet and found an increase in naso-anal length.

According to Bernardis and Patterson^23^ (1968), Lee’s index is an effective tool to determine obesity in animals. This parameter and the animal’s fat mass are related: the higher the Lee index, the greater the animal’s chance of becoming obese.^23^ A Lee index value below 0.30 g/cm is considered normal.^22^ In the present study, the animals in the obese groups obtained a Lee index above 0.30 g/cm, corroborating the data from the literature.

One of the main measures used to assess obesity and its association with other diseases is the BMI. However, BMI only indicates the percentage of mass (lean or fat), and it is necessary to evaluate other parameters to draw a conclusion about obesity. In the present study, the average BMI of the obese (0.68 g/cm^2^) and obese with osteonecrosis (0.69 g/cm^2^) groups was greater than that of the healthy and osteonecrosis groups (0.44 g /cm^2^). These results were similar to previous reports in the literature about diets altering BMI.^22^

Other anthropometric parameters were also evaluated in this study, such as deposits of adipose tissue through the circumference of the abdominal and thoracic regions. We observed an increase in both measures in the obese groups compared to the non-obese groups. A similar study indicated that the accumulation of fat in the thoracic region may be correlated with diabetes.^24^ The increase in the abdominal circumference may also be a predictive factor for cardiovascular complications and is associated with the risk of type 2 diabetes, thus representing a stronger indicator than the BMI.^25^

In the present study, the animals in the obese groups (with and without osteonecrosis) displayed an increase in the three fat deposits compared to animals in the other groups, resulting in an increased adiposity index. Bortolin, et al.^26^ (2018) carried out a study comparing some obesity-inducing diets in an animal model and demonstrated that only the westernized diet caused an increase in these animals’ adiposity index, indicating that the diet choice is decisive. No study in the literature directly relates the adiposity index increase in obese animals and in those with osteonecrosis, as demonstrated in our study. Nascimento, et al.^27^ (2011) conducted a survey with Wistar rats under a high-fat diet (49%) for 15 weeks and observed an increase in their adiposity index by approximately 4%.

Numerous comorbidities can arise in parallel to obesity, including liver disorders, such as steatosis, which is present in approximately 80%–90% of obese adult individuals.^28^ The disease is mainly characterized by the accumulation of triglycerides in hepatocytes. According to Abdelmalek, et al.^29^ (2010), the increased consumption of fructose in humans worsens hepatic steatosis and fibrosis. One of the main components of the diet used in the study was condensed milk, which corresponds to a disaccharide containing glucose and fructose with approximately 53% of sucrose.

In this study, an increase in the liver weight of animals in the obese groups (with and without osteonecrosis) was observed compared to animals in the non-obese groups. In addition, the obese group with osteonecrosis presented an increase in the size of adipocytes, large and dilated vessels (hepatic stasis), and steatosis in their hepatic parenchyma, indicating that the high-glycemia diet may be related to these observations.

In parallel with the obesity induction, osteonecrosis was induced, using bisphosphonates. The normal healing process of the alveolus after the tooth extraction procedure, as well as the alterations in the repair mechanism caused by the use of bisphosphonates, have been studied in rats, and the results are similar to ours.^30^ Macroscopically, the animals in the healthy group displayed clear, smooth, and homogeneous tissue—compatible with healthy gingiva—on the alveolar surface after tooth extraction. On the other hand, the animals of the osteonecrosis, obese, and obese with osteonecrosis groups displayed an absence of epithelial lining in the tooth extraction region, compatible with exposed bone.

Bisphosphonates are a class of medications that act by inhibiting bone resorption. The anti-resorptive effect of these drugs is primarily attributed to their inhibitory action on osteoclasts. Bisphosphonates concentrate in the bones due to their high affinity with hydroxyapatite crystals. During osteoclastic resorption, these drugs are phagocytosed along with the degraded bone matrix by the action of proteases and acids released by these cells. Once phagocytosed, bisphosphonates act within the osteoclasts, impairing their function.^3^

Therefore, bisphosphonates decrease and inhibit bone resorption by inducing osteoclastic apoptosis, inhibiting mature osteoclasts. Bone metabolism involves two activities: deposition and resorption. In deposition, osteoblasts synthesize a matrix that undergoes primary mineralization and a subsequent long process of secondary mineralization. On the other hand, bone resorption is carried out by osteoclasts and involves bone mineral dissolution and catabolism of bone matrix components.^31^ *In vitro* studies have identified that osteoblasts are sensitive to bisphosphonates.^32,33^

In addition to the presence of anucleate osteocytes, specifically in animals from the obese and obese with osteonecrosis groups, bone sequestration was identified, suggesting that obesity is associated with more severe tissue damage. Bone sequestration has been demonstrated in non-obese animals through studies that performed osteonecrosis induction.^34,35^ However, this is the first report of bone sequestration in the mandible of obese animals with osteonecrosis.

The study indicated that, when associated with osteonecrosis, obesity induces an increase in the bone necrosis area, making the disease even more serious. This was confirmed through the morphometric analysis of the necrosis areas in each group. We observed significant differences between the healthy group and the others (osteonecrosis, obese, and obese with osteonecrosis). The necrosis area was greater in the obese with osteonecrosis (290.33 µm^2^) and osteonecrosis (172.83 µm^2^) groups, indicating that, in addition to being efficient, the osteonecrosis induction protocol generated an even larger necrosis area when associated with obesity.

This correlation may have occurred due to some factors, such as the characteristic inflammation of both diseases. Studies by Zandi, et al.^34^ (2016) and Vilarinho, et al.^35^ (2017) pointed to the presence of inflammatory infiltrate in the area of lesions induced by the manifestation of osteonecrosis in the jaws of rats. Bone necrosis can lead to the loss of soft tissue integrity. In addition, the extraction itself causes direct rupture of the soft tissues, which makes the disease even more severe, favoring the prolongation of inflammation in a positive feedback loop, increasing its severity.

Lesclous, et al.^36^ (2009) conducted a study analyzing detailed medical and dental histories of 30 patients with bisphosphonate-associated osteonecrosis of the jaw (ONJ) due to malignant diseases or osteoporosis. Their aim was to examine clinical and histopathological features in an attempt to better understand the ONJ pathogenesis. The study concluded that the clinical extent of the disease was statistically related to the number of inflammatory cells, suggesting that bone necrosis precedes clinical onset and is associated with inflammation or extent of the disease.^36^

In addition to the inflammation in the area of the mandible lesions, we observed inflammation in the adipose tissue of some experimental groups. Through microscopic evaluation, the animals of the healthy and osteonecrosis groups presented adipocytes without inflammatory processes. However, the animals from the obese and obese with osteonecrosis groups presented large adipocytes, and rats from the obese with osteonecrosis group presented large, dilated vessels (blood stasis) in their adipose tissue.

Obesity is a disease characterized by the adipose tissue accumulation, regardless of age, sex, or height, and it is directly associated with inflammation and various chronic non-communicable diseases, thereby influencing body composition. Luz, et al.^12^ (2018) conducted a study with Wistar rats using a high-glycemic index diet and observed an increase in the inflammatory process.

Obesity is linked to inflammation mainly due to the fact that, in obese patients, the circulating level of many cytokines and acute phase proteins associated with inflammation is high. Adipocytes secrete several cytokines and acute phase proteins that, directly or indirectly, increase the production and circulation of factors related to the inflammatory process.^37^ However, understanding the origin of inflammatory markers in obesity is necessary for a better understanding of this correlation.

According to Yudkin, et al.^38^ (1999) inflammatory markers have three possible origins. First, they might be produced by organs other than adipose tissue, such as the liver, in addition to immune cells. Second, white adipose tissue might secrete factors, stimulating the production of inflammatory markers by the liver and other organs. And finally, inflammatory markers might originate from the adipocytes themselves.

This study has limitations, such as technical difficulties during the surgical procedure, for instance, reduced extraction time due to anesthesia-induced apnea and respiratory difficulties (caused by the supine position and manipulation in the oral cavity). There is still a limited amount of research reporting the complications that occur during extractions and detailed descriptions of the technical difficulties involved. Despite these limitations, the specific model used for inducing osteonecrosis allowed for the analysis of MRONJ, similarly to the study conducted by Howie, et al.^39^ (2015).

Limitations in this study also include the difficulty in determining the region of interest for histomorphometric analysis, which enables the description of morphology under physiological or pathological conditions but does not provide information about the entire process dynamics or the mechanisms involved behind the presentation, even with the analysis of serial sections. Another challenge is related to the anatomical complexity of the jaw, which makes it difficult to select homogeneous areas for the application of the technique. Therefore, more evidence is needed to clarify these questions.

Despite the mentioned limitations, animal models play a significant role in studying human diseases, allowing for a better understanding of pathological processes and for the discovery of new treatments. The literature describes animal models to comprehend the pathophysiology of MRONJ. Rats and mice are frequently used in these models due to their small size, which facilitates handling, their genetic similarity to humans, and their high reproductive capacity. These characteristics make rodents a suitable choice for studying this condition.^40^

The findings in this study are important for the exploration of new strategies in dental treatment of cases involving oral cavity issues that require interventions in obese patients who are also using bisphosphonates, regardless of the reason. It is important to emphasize the need for conducting studies on these topics, as understanding the origins of the pathophysiological mechanisms of diseases is crucial to seek strategies not only for their prevention but also for their cure.

## Conclusion

The influence of obesity on the development of bisphosphonate-induced osteonecrosis was analyzed using animal models that provided important insights into both diseases.

The induction and standardization of the obesity and osteonecrosis model in Wistar rats were carried out, evaluating the parameters described in the literature that supported the obtained results.

The main macroscopic and microscopic findings regarding the mandible were: the animals in the healthy group showed preserved epithelial lining, while the animals in the groups with osteonecrosis, obese, and obese with osteonecrosis exhibited absence of epithelial lining. The mean necrosis areas were smaller in the healthy and osteonecrosis groups and larger in the obese with osteonecrosis and obese groups.

Regarding the analyzed adipose tissue, the mean adipocyte size was smaller in the healthy and osteonecrosis groups and larger in the obese and obese with osteonecrosis groups, and only the latter group presented dilated vessels corresponding to blood stasis in adipose tissue.

It was observed that all analyzed osteonecrosis parameters increased for the obese and obese with osteonecrosis groups, suggesting a potential influence of obesity on these results. However, further studies are needed to confirm this possible relationship, especially involving the molecular mechanisms and inflammatory processes described.
